# Enhancement of Force Generated by Individual Myosin Heads in Skinned Rabbit Psoas Muscle Fibers at Low Ionic Strength

**DOI:** 10.1371/journal.pone.0063658

**Published:** 2013-05-15

**Authors:** Haruo Sugi, Takahiro Abe, Takakazu Kobayashi, Shigeru Chaen, Yoshiki Ohnuki, Yasutake Saeki, Seiryo Sugiura

**Affiliations:** 1 Department of Physiology, School of Medicine, Teikyo University, Tokyo, Japan; 2 Department of Electronic Engineering, Shibaura Institute of Technology, Tokyo, Japan; 3 Department of Integrated Sciences in Physics and Biology, College of Humanities and Sciences, Nihon University, Tokyo, Japan; 4 Department of Physiology, School of Dentistry, Tsurumi University, Yokohama, Japan; 5 Graduate School of Frontier Sciences, University of Tokyo, Tokyo, Japan; Cinvestav-IPN, Mexico

## Abstract

Although evidence has been presented that, at low ionic strength, myosin heads in relaxed skeletal muscle fibers form linkages with actin filaments, the effect of low ionic strength on contraction characteristics of Ca^2+^-activated muscle fibers has not yet been studied in detail. To give information about the mechanism of muscle contraction, we have examined the effect of low ionic strength on the mechanical properties and the contraction characteristics of skinned rabbit psoas muscle fibers in both relaxed and maximally Ca^2+^-activated states. By progressively decreasing KCl concentration from 125 mM to 0 mM (corresponding to a decrease in ionic strength **μ** from 170 mM to 50 mM), relaxed fibers showed changes in mechanical response to sinusoidal length changes and ramp stretches, which are consistent with the idea of actin-myosin linkage formation at low ionic strength. In maximally Ca^2+^-activated fibers, on the other hand, the maximum isometric force increased about twofold by reducing KCl concentration from 125 to 0 mM. Unexpectedly, determination of the force-velocity curves indicated that, the maximum unloaded shortening velocity V_max_, remained unchanged at low ionic strength. This finding indicates that the actin-myosin linkages, which has been detected in relaxed fibers at low ionic strength, are broken quickly on Ca^2+^ activation, so that the linkages in relaxed fibers no longer provide any internal resistance against fiber shortening. The force-velocity curves, obtained at various levels of steady Ca^2+^-activated isometric force, were found to be identical if they are normalized with respect to the maximum isometric force. The MgATPase activity of muscle fibers during isometric force generation was found not to change appreciably at low ionic strength despite the two-fold increase in Ca^2+^-activated isometric force. These results can be explained in terms of enhancement of force generated by individual myosin heads, but not by any changes in kinetic properties of cyclic actin-myosin interaction.

## Introduction

It is generally believed that the mechanical activity of vertebrate skeletal muscle is regulated by binding of Ca^2+^ to troponin on the thin filaments, which produces a shift in the position of tropomyosin on the thin filaments. This tropomyosin position shift in turn regulates attachment-detachment cycle between myosin heads extending from the thick filaments and actin in the thin filaments [1]. Contrary to this view, however, in vitro myosin head binding to regulated actin filaments, containing the troponin-tropomyosin system, takes place to the same extent irrespective of presence or absence of Ca^2+^ at low ionic strength [2], [3]. In accordance with the above in vitro biochemical results, relaxed rabbit psoas muscle fibers at low ionic strength show substantial stiffness in response to rapid stretches [4], as well as X-ray diffraction patterns analogous to that of rigor state [5], [6]. These results indicate that, in relaxed fibers, myosin heads form linkages with actin at low ionic strength. Up to the present time, however, the physiological implication of the actin-myosin linkages at low ionic strength still remains unclear. On the other hand, the magnitude of Ca^2+^-activated isometric force has been known to increase with decreasing ionic strength in mechanically skinned frog muscle fibers [7], [8], [9]. The results on frog muscle fibers are, however, complicated by incomplete relaxation of the fibers due to residual force that persists in relaxing solution and is variable depending on both the quality of fiber preparation and experimental conditions used. The present experiments were undertaken to give information about the mechanism of muscle contraction by thoroughly examining the effect of low ionic strength on mechanical properties and contraction characteristics in skinned rabbit psoas muscle fibers at rest and during Ca^2+^-activated mechanical activity.Here we report that, when the KCl concentration was reduced progressively from 125 mM to 0 mM (corresponding to a decrease in ionic strength **µ**from 170 mM to 50 mM), (1) relaxed fibers show mechanical responses to sinusoidal length changes and ramp stretches consistent with possible formation of actin-myosin linkages, (2) the magnitude of steady Ca^2+^-activated isometric force increases about twofold, (3) Ca^2+^-activated fibers can be made to relax completely in relaxing solution without any detectable residual force, (4) the maximum unloaded shortening velocity V_max_ of Ca^2+^-activated fibers remains unchanged, suggesting that the actin-myosin linkages, detected in relaxed fibers, disappears rapidly on Ca^2+^ activation, so that the linkages no longer provide any internal resistance against fiber shortening, and (5) the MgATPase activity during steady isomeric force generation does not change appreciably, despite the twofold increase of the Ca^2+^-activated isometric force. These results can most readily be explained in terms of the enhancement of force generated by individual myosin heads at low ionic strength. Possible mechanisms of the enhancement of force generating ability in individual myosin heads are discussed in connection with the electrostatic nature of actin-myosin interaction in muscle.

## Materials and Methods

### Skinned Muscle Fiber Preparation

White male rabbits weighing 2 to 2.5 kg were killed by injection of sodium pentobarbital (50 mg/kg) into the ear vein, and psoas muscles were dissected from the animals. The animals were treated in accordance with the Guiding Principles for the Care and Use of Animals in the Field of Physiological Sciences, published by the Physiological Society of Japan. The protocol was approved by the Teikyo University Animal Care Committee (protocol #07–050). Glycerol-extracted muscle fiber strips were prepared from the psoas muscle as described by Sugi et al. [10]. Single muscle fibers (diameter, 50–100 **µ**m) were dissected from the fiber strips, and mounted horizontally in an experimental chamber (40 **µl**) between a force transducer and a servomotor by gluing both ends with collodion. The servomotor contained a displacement transducer (differential capacitor) sensing the motor arm position. Further details of the experimental apparatus have been described elsewhere [10]. The fibers were kept at the slack length Lo (∼3 mm) at a sarcomere length of 2.4 **µ**m, measured with optical diffraction by He-Ne laser light. Relaxing solution contained 125 mM KCl, 4 mM MgCl_2_, 4 mM ATP, 4 mM EGTA, 20 mM Pipes (pH 7.0). Contracting solution was prepared by adding 4 mM CaCl_2_ to relaxing solution to maximally activate the fibers. The ionic strength of experimental solutions was varied by reducing KCl concentration. The experiments were mostly performed at 2–3°C, since at temperatures of 15–20°C, the high Ca^2+^-activated isometric force attained at low ionic strength, tended to give damage to the fibers; after reaching a peak, the isometric force did not stay constant but fell abruptly, and the fibers were deteriorated. At 2–3°C, the maximum Ca^2+^-activated force at low ionic strength was reduced to less than 50% of that at 15–20°C, and gave no appreciable damage to the fibers. Solutions were exchanged by draining and refilling the experimental chamber with micropipettes.

### Measurement of Muscle Fiber Stiffness

Muscle fiber stiffness in both relaxed and maximally Ca^2+^-activated fibers was measured continuously by applying small sinusoidal length changes (frequency, 2 kHz) with the servomotor and recording the resulting force changes [10], [11]. Stiffness values were obtained from the ratio of force change versus length change. The peak-to-peak amplitude of sinusoidal length changes was 0.5% of Lo for relaxed fibers (6 nm/half sarcomere) and 0.2% of Lo for Ca^2+^-activated fibers (2.4 nm/half sarcomere). In the case of relaxed fibers, they were slightly stretched beyond Lo prior to the stiffness measurements, to obtain sinusoidal force changes around a steady level of resting force (>0.03 mN). The force changes were recorded in pairs; one with and one without sinusoidal length changes. Electronic subtraction of the records were made in a digital oscilloscope to obtain force records containing only the sinusoidal component [11].

### Determination of Force-Velocity Relation in Ca^2+^-Activated Fibers

The servomotor system operated either in the length clamp mode or in the force control mode [10], [12]. First, the servomotor system was in the length clamp mode, so that the fiber (sarcomere length, 2.4 µm) contracted isometrically in response to contracting solution. When the fiber developed steady isometric force, the servomotor system was switched to the force control mode, and a ramp decrease in force from the steady isometric force to zero(complete in 0.15–0.3 s) was applied to the fiber. The resulting fiber shortening was recorded together with the ramp decrease in force. This method enabled us to obtain instantaneous relation between the velocity of fiber shortening and the force ( = load) generated by the fiber, without making the fiber to shorten repeatedly under various isotonic load. The force-velocity curve thus obtained was displayed on an X–Y chart recorder. Further details of the method have been described elsewhere [10], [12].

### Measurement of MgATPase Activity

MgATPase of small fiber bundles consisting of 2–3 muscle fibers was recorded by the decrease of NADH during ATP hydrolysis during Ca^2+^-activated isometric force development [10] at 10°C, to obtain clear ATPase records suitable for analysis. The fibers were mounted in the sample compartment (45 **µ**l) of a dual wavelength spectrophotometer (Nihon Bunko) with a sample monochrometer at 340 nm and a reference monochrometer at 400 nm, so that the decrease of NADH was recorded from the difference in absorbance between 340 and 400 nm. One end of the fibers was clamped while the other end was connected to the force transducer. To both relaxing and contracting solutions, 0.25 mM NADH, 1.25 mM phosphoenolpyruvate, 50 units/ml pyruvate kinase, 50 units/ml lactic dehydrogenase, 10 mM NaN_3_, 50 **µ**M quercetin, 1 **µ**g/ml oligomycin were added. Solutions in the compartment were constantly stirred with a magnetic stirrer.

## Results

### Effect of Low Ionic Strength on Stiffness in Relaxed Muscle Fibers

**Figure 1:**
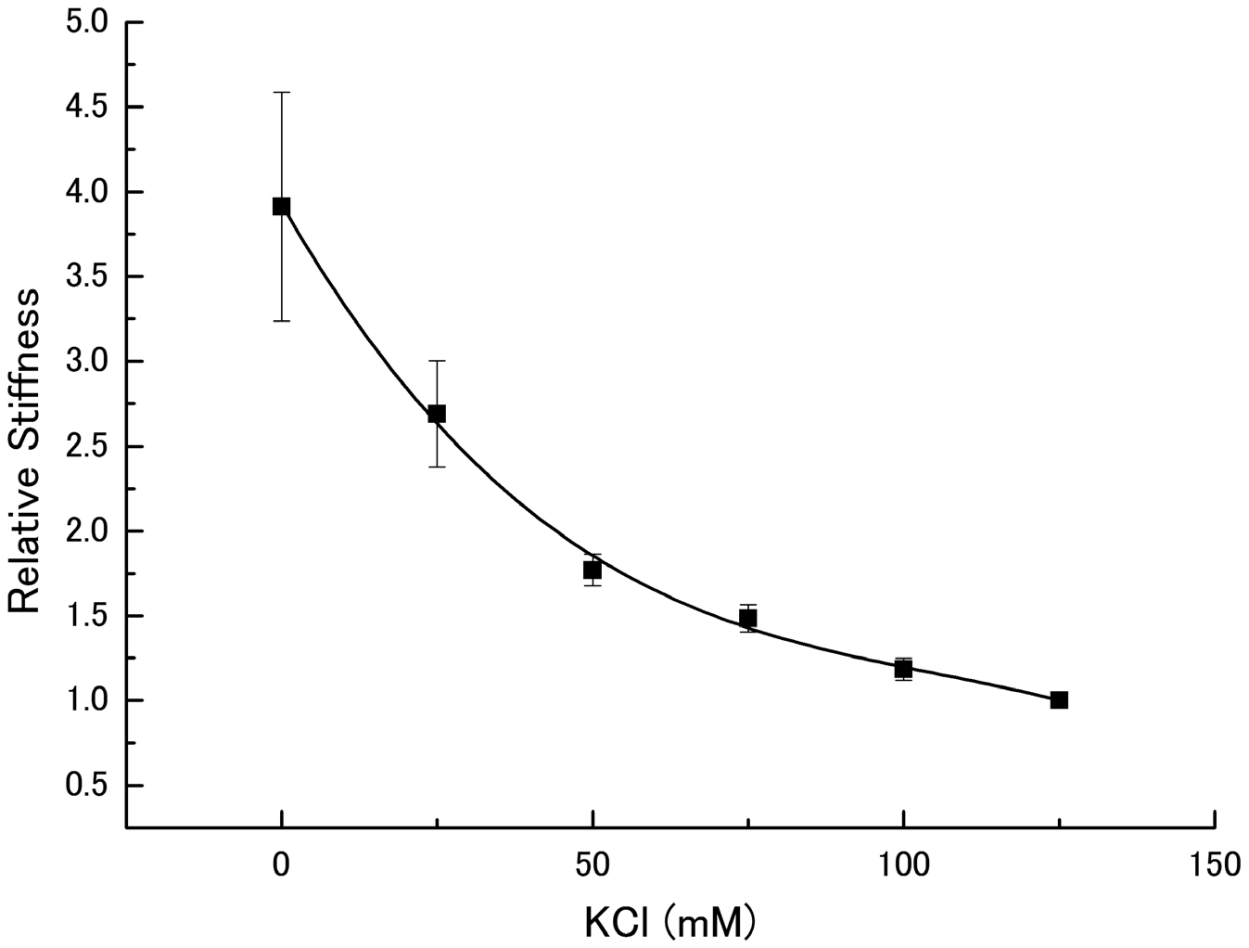
Dependence of relaxed muscle fiber stiffness on ionic strength. Stiffness values of relaxed muscle fibers were obtained by applying sinusoidal length changes (peak-to-peak amplitude, 0.5% of Lo; frequency, 2 kHz), and the values relative to the value at 125 mM KCl are plotted against KCl concentration of experimental solution. Vertical bars represent S.E.M. (n = 5). The stiffness measurements were made in a random sequence.

The stiffness in relaxed muscle fibers was measured when KCl concentration of relaxing solution was progressively decreased from the standard value of 125 mM to 0 mM (corresponding to reduction of ionic strength **μ** from 170 mM to 50 mM). As shown in Figure 1, the stiffness of relaxed fibers increased steeply with decreasing KCl concentration. The value of stiffness at 0 mM KCl was about four times larger than that at the standard KCl concentration of 125 mM.

**Figure 2:**
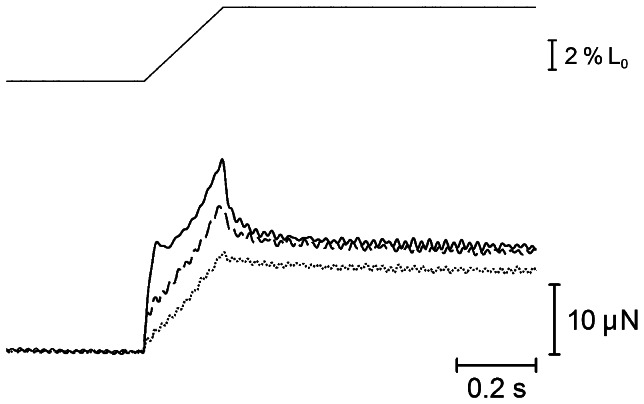
Force responses in relaxed muscle fibers in to ramp stretches. Ramp stretches (amplitude, 5% of Lo, complete in 0.2 s) were applied at KCl concentrations of 0 mM (solid line), 50 mM (broken line), and 125 mM (dotted line). Note the marked abrupt force increase at the beginning of stretch at 0 mM KCl.

To obtain information about the underlying mechanism of the increased stiffness at low ionic strength, ramp stretches (5% of Lo, complete in 0.2 s; stretch velocity, 300 nm/s/half sarcomere) were applied to relaxed fibers at various KCl concentrations. As shown in Figure 2, the force increased to a peak, which was reached at the end of stretch, and then decreased to a steady level. At KCl concentrations <50 mM, the force response to stretch exhibited a marked abrupt increase at the beginning of stretch, and the initial abrupt force increase was sometimes followed by a transient force decrease.

### Effect of Low Ionic Strength on Stiffness and Isometric Force Generation in Ca^2+^-Activated Fibers

**Figure 3:**
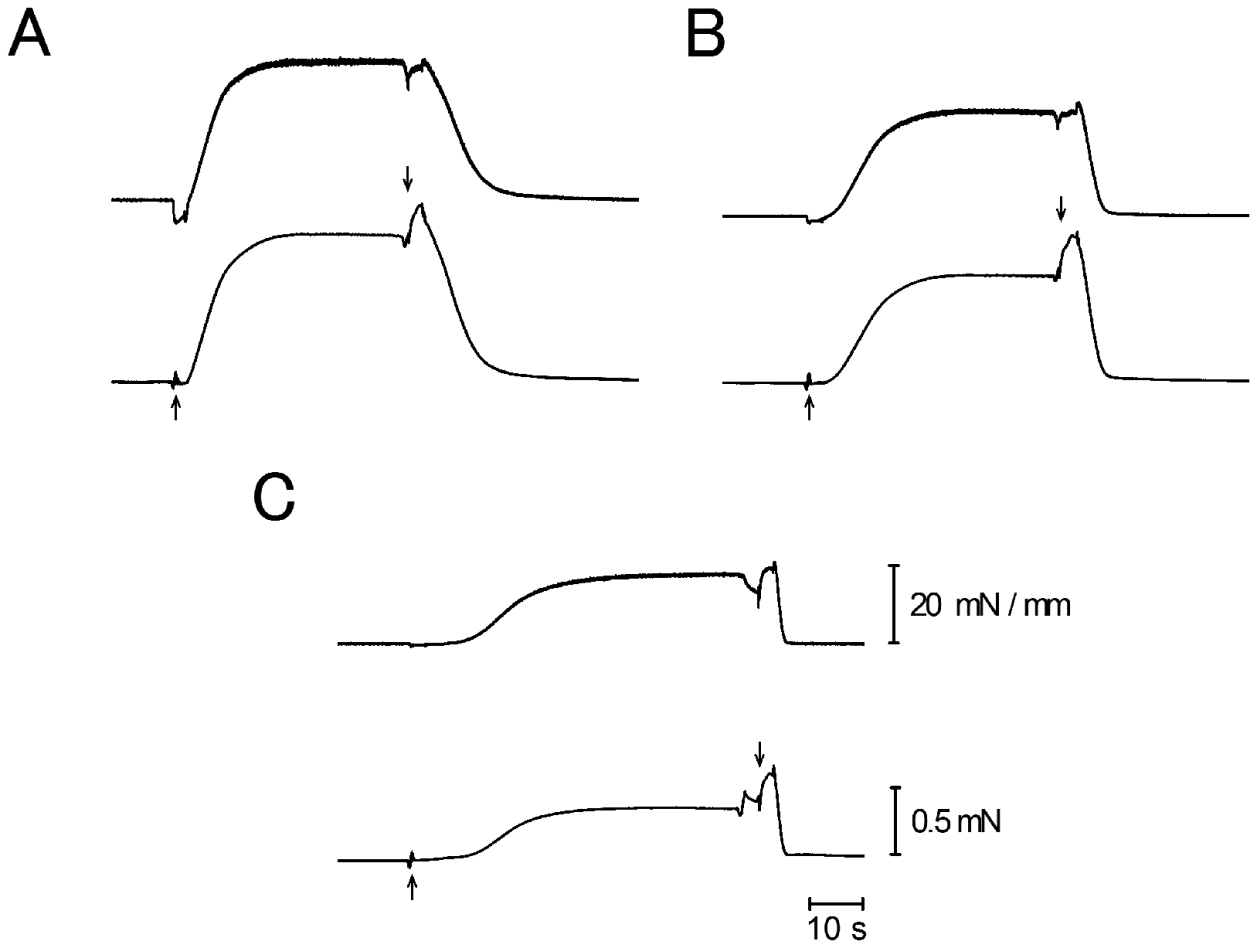
Stiffness and force changes during the isometric force development. Simultaneous recordings of stiffness (upper records) and force (lower records) during the development of steady isometric force in contracting solution. The KCl concentration was 0 mM, 50 mM and 125 mM in records A, B and C, respectively. Upward and downward arrows indicate time of application and removal of contracting solution, respectively. The force increment on returning the fiber to relaxing solution is an artifact accompanying solution exchange procedure. The same explanation applies to force records shown in Figure 7.

**Figure 4:**
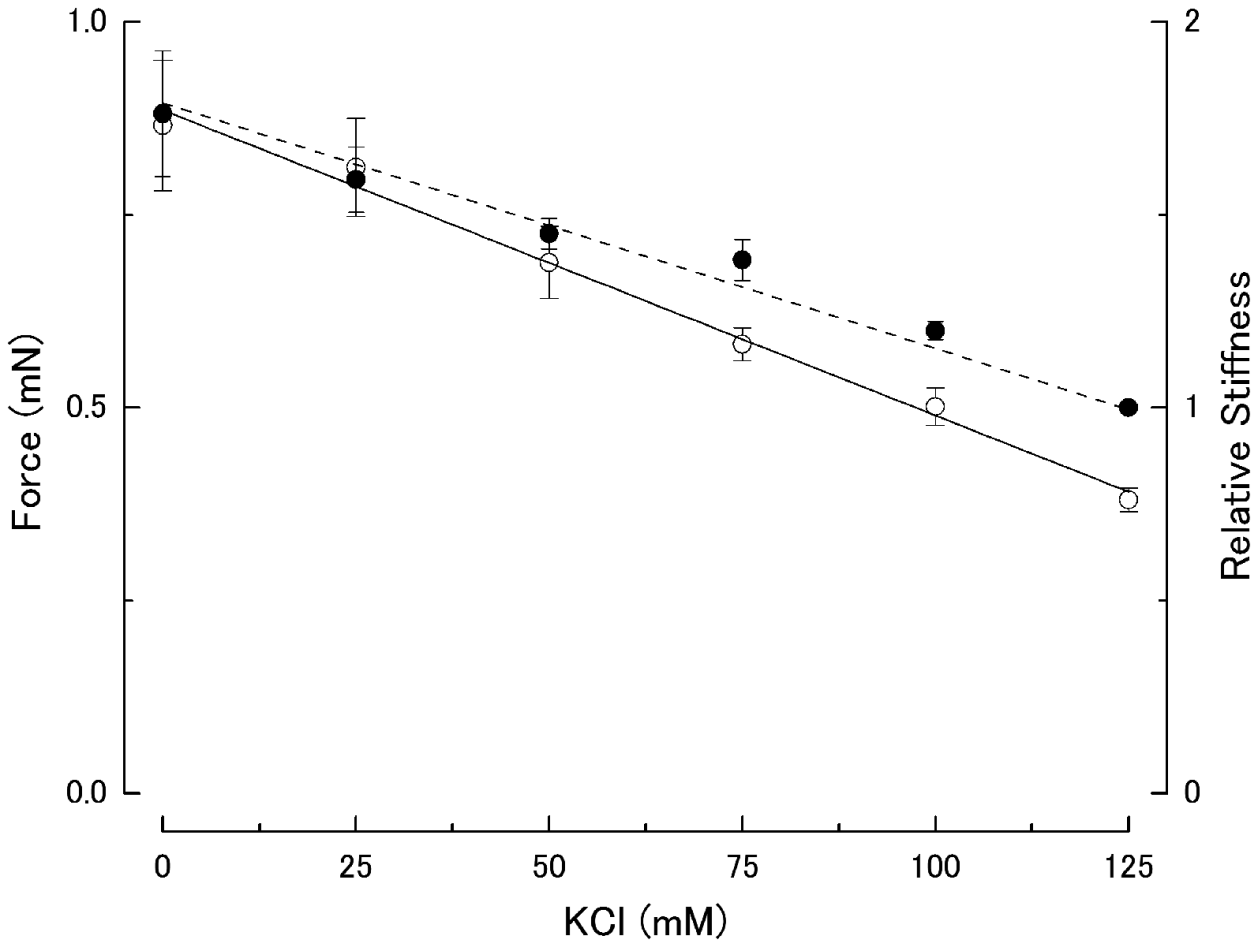
Dependence of steady isometric force and stiffness on ionic strength. Steady Ca^2+^-activated isometric force (open circles) and corresponding stiffness (filled circles) are plotted against KCl concentration. Vertical bars represent S.E.M. (n = 5). Stiffness values are expressed relative to the value at 125 mM KCl.

When a muscle fiber was maximally activated in contracting solution at the standard KCl concentration (125 mM), it generated steady Ca^2+^-activated isometric force amounting 50–60 kN/m^2^. Figure 3 shows typical examples of force and stiffness records at different KCl concentrations. On Ca^2+^-activation, both the force and stiffness increased nearly in parallel with each other to reach their respective steady values [10], [11]. In agreement with previous studies on mechanically skinned frog muscle fibers [7], [8], [9], the magnitude of steady Ca^2+^-activated isometric force increased about twofold when the KCl concentration was decreased from 125 mM to 0 mM. Contrary to the previous studies, however, the Ca^2+^-activated fibers could be made to relax completely in relaxing solution without any detectable residual force. Figure 4 shows dependence of the steady Ca^2+^-activated isometric force and the corresponding stiffness on the KCl concentrations below 125 mM, obtained from five different fibers activated at different KCl concentrations in a random sequence. As reported by Gulati & Podolsky [9], the steady Ca^2+^-activated isometric force increased almost linearly with decreasing KCl concentration from 125 mM to 0 mM.

### Effect of Low Ionic Strength on Force-Velocity Relation in Ca^2+^-Activated Fibers

**Figure 5:**
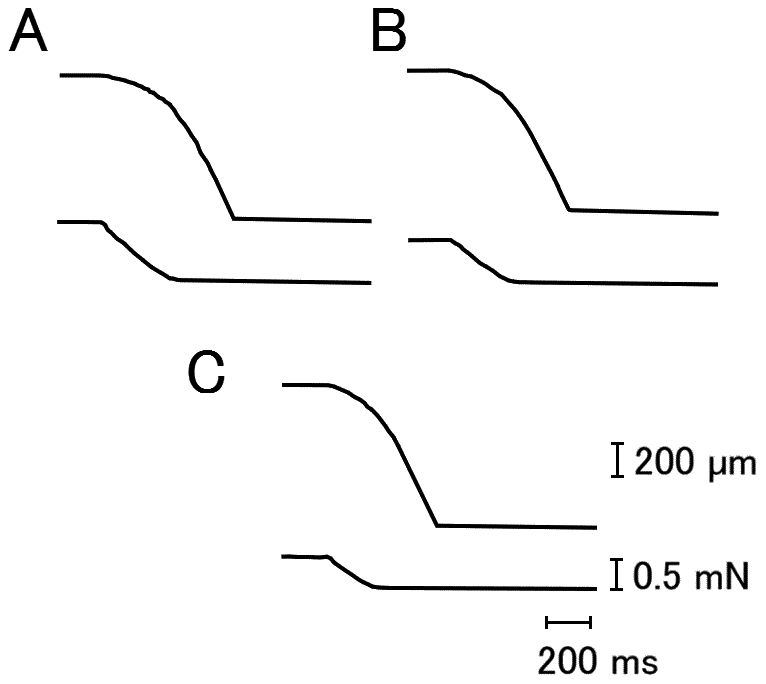
Fiber length changes to ramp decreases in force applied at steady isometric force. Ramp decreases in force from the level of steady Ca^2+^-activated isometric force to zero (complete in 0.15–0.3 s) were applied to one and the same muscle fiber at KCl concentrations of 0 mM (A), 50 mM (B), and 125 mM (C).

**Figure 6:**
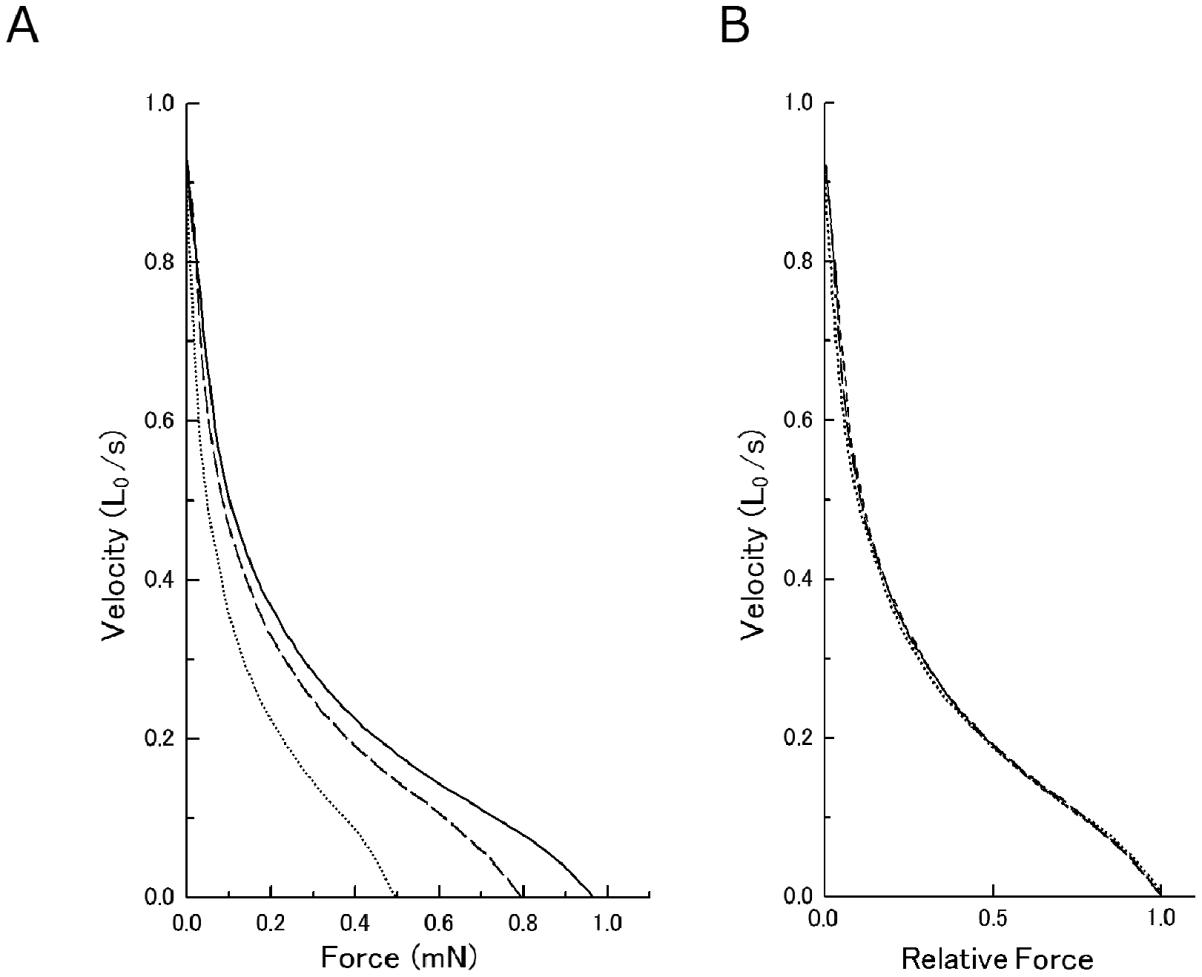
Effect of low ionic strength on force-velocity curves of Ca^2+^-activated muscle fibers. (A) The force-velocity curves obtained at 0 mM KCl (solid line), 50 mM KCl (broken line), and 125 mM KCl (dotted line). Note that the maximum velocity of shortening remains unchanged irrespective of the steady isometric forces attained. (B) The same force-velocity curves, in which forces are expressed relative to their steady isometric forces. Note that the curves are identical in shape.

Figure 5 presents examples of fiber length and force changes when ramp decrease in force was applied during the steady isometric force generation in Ca^2+^-activated fibers (initial sarcomere length, 2.4 µm). Force-velocity curves were constructed from these records at various KCl concentrations below 125 mM. As can be seen in Figure 6A, the force-velocity curves showed a hump in the high load region, resembling closely the double-hyperbolic force-velocity curve in intact single muscle fibers [13]. The maximum unloaded shortening velocity (0.8–1.0 Lo/s, 960–1200 n/s/half sarocomere), determined by the point at which the curve intersects with the velocity axis, remained unchanged irrespective of the magnitude of isometric force attained at various KCl concentrations, so that the curves were identical if the force values were normalized with respect to the steady values (Figure 6B). Similar results were obtained on five other fibers examined. This finding indicates that the increased resting fiber stiffness (Figure 1) at low ionic strength does not provide any internal resistance against myofilament sliding.

### Effect of Low Ionic Strength on MgATPase Activity of Ca^2+^-Activated Fibers

**Figure 7:**
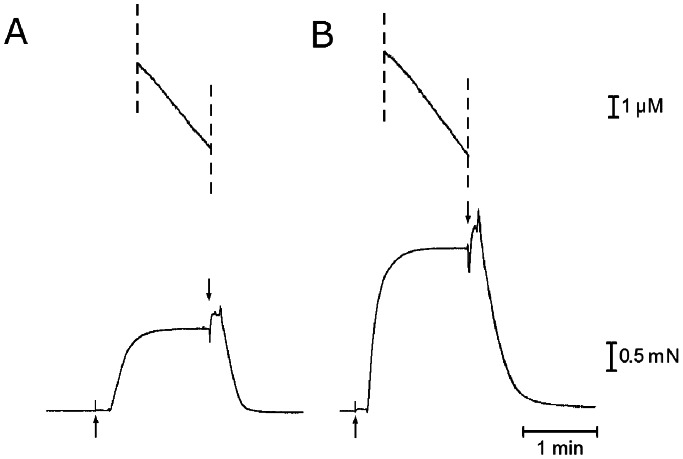
Simultaneous recordings of MgATPase activity and isometric force in maximally Ca^2+^-activated muscle fibers. MgATPase activity (upper traces) and isometric force (lower traces) obtained from one and the same fiber at 125 mM KCl (A) and at 0 mM KCl (B). Note that the slope of MgATPase records does not differ markedly between the two records, despite the twofold increase of isometric force.

Fig.7 shows an example of simultaneous recording of MgATPase activity (upper traces) and Ca^2+^-activated isometric force (lower traces). Although the Ca^2+^-activated steady isometric force increased about twofold by reducing the KCl concentration from 125 to 0 mM, the MgATPase activity of the fibers, estimated from the constant slope of the ATPase race, showed no marked changes. To ascertain whether the MgATPase changes at low ionic strength, we compared the MgATPase activity at 125 mM KCl (A_125_) with that at 0 mM KCl (A_0_) on ten different fiber bundles. To minimize the effect of structural instability of skinned fibers (Brenner, 1998), simultaneous recording of force and MgATPase activity for each preparation were limited to two times; one recording at 125 mM KCl and the other recording at 0 mM KCl in a random sequence. In six out of ten preparations, A_0_ was larger than A_125_, while A_125_ was larger than A_0_ in the rest four preparations. The average values of A_125_ and A_0_ were 0.53±0.15 mM/s and 0.56±0.18 mM/s (mean ±SD, n = 10), respectively. These results may be taken to indicate that the MgATPase of the fibers did not change significantly despite the twofold increase of the isometric force. The values of MgATPase activity obtained in the present study are consistent with those reported previously [14], [15], if differences in experimental conditions are taken into consideration.

## Discussion

### Formation of Actin-Myosin Linkages in Relaxed Muscle Fibers at Low Ionic Strength

In relaxed rabbit psoas muscle fibers, evidence has been presented that myosin heads extending from the thick filaments form linkages with actin in the thin filaments [4], [6], [16]. If the ionic strength is reduced progressively, the intensity of *11* equatorial reflection increases while that of *10* reflection decreases, indicating gradual transfer of mass from the thick to thin filaments with decreasing ionic strength, and these intensity changes appear not to be influenced by changes in filament-lattice spacing [5]. The gradual increase of relaxed muscle fiber stiffness with decreasing ionic strength (Figure 1), observed in the present study, would reflect an increasing population of myosin heads forming linkages with actin. In accordance with these reports, the transient abrupt increase of force and stiffness in relaxed fibers at the beginning of ramp stretch, applied at low KCl concentrations (Figure 2), might be explained as being due to synchronized distortion of actin-myosin linkages [11]. The increasing variation of muscle fiber stiffness values at low ionic strength, as indicated by increasing SEM values, might result from a wide range of variation in the proportion of myosin heads forming linkages with actin at low ionic strength.

### Evidence for Quick Breaking of Actin-Myosin Linkages in Relaxed Fibers on Ca^2+^-Activation

Although the nature and physiological significance of the actin-myosin linkages at low ionic strength remains obscure, the present study has made it clear that the actin-myosin linkages, formed at low ionic strength, are broken quickly when the fibers are activated with Ca^2+^. The present finding that the maximum unloaded shortening velocity V_max_ remains unchanged at low pH (Figure 6) indicates that the actin-myoin linkages and/or increased relaxed fiber stiffness (Figures 1 and 2) at low ionic strength no longer serve as increased internal resistance against fiber shortening after structural changes accompanying Ca^2+^-activation. As shown in the figure, the force-velocity curves obtained at various ionic strengths ≤125 mM were identical in shape when normalized with respect to the maximum isometric force attained at various ionic strengths. This implies that kinetic properties of attachment-detachment cycle between myosin head and actin are not influenced by decrease in ionic strength, again indicating complete breaking of actin-myosin linkages in Ca^2+^-activated fibers. A possible explanation, which can be tested experimentally in future, may be that, on binding of Ca^2+^ with troponin, the resulting change in position of tropomyosin around the thin filaments not only initiates actin-myosin interaction cycle, but also quickly break actin-myosin linkages existing in relaxed fibers at low ionic strength.

### Evidence for Increased Isometric Force Generated by Individual Myosin Heads at Low Ionic Strength

The main findings obtained in the present study were; (1) the maximum unloaded shortening velocity of Ca^2+^-activated fibers V_max_ remained unchanged at low ionic strength (Figure 6), despite the increased relaxed fiber stiffness (Figures 1 and 2), and (2) the MgATPase activity of Ca^2+^-activated fibers did not change significantly at low ionic strength, though the Ca^2+^-activated isometric force increased twofold (Figures 3, 6 and 7 ). It is difficult to account for these results by changes in kinetic properties of attachment-detachment cycle between myosin head and actin on the basis of the Huxley contraction model [17]. A most straightforward explanation for the present results may be that, at low ionic strength, the force generated by individual myosin heads increases up to twofold, while the kinetics of the actin myosin interaction coupled with ATP hydrolysis remain unchanged. This explanation implies that the rate of attachment-detachment cycles between myosin head and actin, each coupled with hydrolysis of one ATP molecule, remains unchanged when the force generated by individual myosin head increases at low ionic strength; meanwhile, the value of V_max_ remains constant at low ionic strength, since it is determined only by the rate of the attachment-detachment cycle.

This idea is supported by the result that the force-velocity curves recorded at various ionic strengths were identical in shape, so that the curves were scaled according to the maximum Ca^2+^-activated isometric forces (Figure 6 ), since the force-velocity relation reflects kinetic properties of actin-myosin attachment-detachment cycle. The above explanation implies that, in the present experimental conditions, the number of myosin heads involved in isometric force generation remains constant, and the steady stiffness during the steady isometric force generation only reflects strain of passive sarcomeric elastic structures. If, on the other hand, the number of myosin head, producing a constant force, is assumed to increase up to twofold at low ionic strength, it is necessary to assume a 50% reduction of the MgATPase activity for each myosin head at low ionic strength, which is unlikely and difficult to explain.

In the Huxley contraction model [16], the myofilaments were assumed to be rigid, so that muscle fiber stiffness was taken to indicate the number of myosin heads attached to actin. However, this interpretation of the stiffness is no longer valid after the discovery that the myofilaments have finite elasticity [18], [19]. The sarcomeric elastic structures, contributing to muscle fiber stiffness may involve myosin heads, myofilaments, and other elements (e.g. titin-related structures). During the period of steady isometric force generation, however, the force generated by myosin heads is balanced with the force arising from overall strain of these passive elastic structures. Therefore, if attention is focused on steady stiffness values during steady isometric force generation, their parallel relation (Figure 4) may be taken to imply that the sarcomeric elasic structures, connected in series with force-generating myosin heads, behave like a linear spring during steady isometric force generation.

The conclusion that the force generated by individual myosin heads increases markedly at low ionic strength is supported by our recent observation using the gas environmental chamber (EC), which enables us to record ATP-induced myosin head movement in hydrated thick filaments electron microscopically [20], [21], [22]. By this novel method, we already succeeded in recording ATP-induced myosin head power stroke in the thick filaments, which are surrounded by the thin filaments forming rigor linkages with myosin heads. When only a small fraction of myosin heads are activated with ATP, the resulting myosin head movement should takes place in a nearly isometric condition; namely, each myosin head performes its power stroke by stretching adjacent sarcomeric strautures, as the overall filament sliding is suppressed by dominant actin-myosin rigor linkages. As a result, the amplitude of myosin head movement, recorded by the EC, was small, being about 3 nm [23]. Moreover, we have recently found that the amplitude of ATP-induced power stroke in individual myosin heasds increases to 4–5 nm, when the KCl concentration of experimental solution is reduced from 125 to <20 mM [24]. This finding can be accounted for most readily in terms of enhanced of force gerarated by individual myosin heads at low ionic strength.

### Evidence for the Electrostatic Mechanism of Myosin Head Force Generation

In our EC experiments [23], individual myosin heads are position-marked by a monoclonal antibody directed to the junctional peptide between 50- and 20-kDa segments of myosin heavy chain [25]. Since the junctional peptide is located in between the two main actin binding sites of myosin heads [1], it seems clear that the antibody attached to the junctional peptide completely covers the two actin binding sites, so that formation of rigor linkages between actin and myosin head is no longer possible. If this explanation is correct, the result of our EC experiments, that individual myosin heads can perform ATP-induced power stroke [23], [24] may be taken to imply that, in the actin-myosin interaction cycle taking place in muscle, myosin heads do not pass through rigor configurations, which is generally believed to exist in the actomyosin ATPase reaction steps [26]. This idea is supported by our recent experiments that this antibody has no effect on both ATP-dependent vitro actin-myosin sliding and Ca^2+^-activated muscle fiber contraction [27]. It seems therefore possible that the so-called strong actin-myosin linkages, responsible for muscle force generation, may be a strong electrostatic attractive force between myosin heads and actin filaments. Since reduction of ionic strength is expected to decrease ionic concentration at the actin-myosin head interface to result in an increased electrostatic attractive forces between myosin and actin, the present results that the force generated by individual myosin heads is enhanced at low ionic strength strongly suggest the electrostatic mechanism of myosin head force generation in muscle.

Concerning the possible electrostatic mechanism in muscle contraction, a novel actin binding site in the myosin head has recently been discovered by Várkuti et al. [28]. This binding site is related to the process of actin activation of myosin head ATPase activity, and is therefore called the activation loop. The ativation loop contains a positively charged amino acid residue, and seems to interact with the negatively charged N-terminal peptide of actin by some electrostatic mechanism. It seems possible that, at low ionic strength, the interaction between the activation loop of myosin and the terminal peptide of actin is modified to result in enhancement of isometric force, though a large gap exists between actomyosin in solution and myofilament lattice of the thick and thin filaments. Much more experimental work is necessary to clarify molecular mechanism of muscle contraction.
